# Upscaling of Carbon-Based Perovskite Solar Module

**DOI:** 10.3390/nano13020313

**Published:** 2023-01-12

**Authors:** Maurizio Stefanelli, Luigi Vesce, Aldo Di Carlo

**Affiliations:** 1 CHOSE—Centre for Hybrid and Organic Solar Energy, Department of Electronic Engineering, University of Rome “Tor Vergata”, Via del Politecnico 1, 00133 Rome, Italy; 2 ISM-CNR, Istituto di Struttura della Materia, Consiglio Nazionale delle Ricerche, via del Fosso del Cavaliere 100, 00133 Rome, Italy

**Keywords:** perovskite solar cells, upscaling, carbon counter electrode, module

## Abstract

Perovskite solar cells (PSCs) and modules are driving the energy revolution in the coming photovoltaic field. In the last 10 years, PSCs reached efficiency close to the silicon photovoltaic technology by adopting low-cost solution processes. Despite this, the noble metal (such as gold and silver) used in PSCs as a counter electrode made these devices costly in terms of energy, CO_2_ footprint, and materials. Carbon-based perovskite solar cells (C-PSCs) and modules use graphite/carbon-black-based material as the counter electrode. The formulation of low-cost carbon-based inks and pastes makes them suitable for large area coating techniques and hence a solid technology for imminent industrialization. Here, we want to present the upscaling routes of carbon-counter-electrode-based module devices in terms of materials formulation, architectures, and manufacturing processes in order to give a clear vision of the scaling route and encourage the research in this green and sustainable direction.

## 1. Introduction

In the last decade, perovskite has earned a lot of attention in solar technology as a photoactive material due to its high carrier mobility, ambipolar transport properties, and bandgap tunability [1,2,3]. Since its discovery in 2009 [3], the perovskite solar cell (PSC) technology has reached a power conversion efficiency of 25.7% for the single junction and 31.25% in tandem with silicon [4,5]. The excellent optoelectronic and light-absorbing properties along with easy and low-cost fabrication are leading this technology to be industrialized and able to supplant the well-established silicon photovoltaics [1].

**Figure 1:**
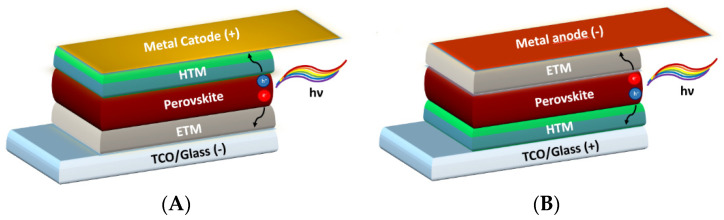
(**A**) Direct/n-i-p and (**B**) inverted/p-i-n perovskite solar cell.

PSCs are made on top of a glass/plastic substrate coated with a Transparent Conductive Oxide (TCO) that works as a front electrode. The absorber is sandwiched between p-type (HTM, Hole Transporting Material) and n-type (or ETM, Electron Transporting Material) semiconductors to improve the charge extraction from perovskite. A top metal electrode (Au, Ag, Cu) completes the device. Perovskite devices could adopt direct (n-i-p) or inverted (p-i-n) configuration (Figure 1A,B). n-i-p or p-i-n junctions are equally used, and which is the most efficient and stable configuration is still under debate in the scientific community [6,7,8].

The n-i-p configuration could be divided into two classes that differ in the ETM materials and the relative structure. The mesoscopic structure employs two layers (compact and mesoporous) of titanium oxide and was used for the first time in DSSC (Dye-sensitized Solar Cell) technology, then transferred to solid-state perovskite devices [4,9,10]. The compact TiO_2_ (c-TiO_2_) works as an electron-blocking layer (EBL) and avoids recombination pathways of photogenerated electrons from perovskite, while the mesoporous TiO_2_ grants the electron transport to the BL and increases the surface contact with perovskite. The planar n-i-p configuration is made by a thin film of different metal oxide (e.g., tin oxide) deposited directly on top of the TCO [11,12,13,14]. The state-of-the-art HTM molecule is the 2,2′,7,7′-tetrakis (*N,N*-di-methoxy-phenylamine)-9,9′-spirobifluorene (spiro-OMeTAD), which provides high efficiencies. Polytriarylamine derivatives (e.g., PTAA) and other highly conjugated organic cores are also used [7,15,16,17]. A gold metal electrode is evaporated on top of the device for its excellent conductibility and bandgap alignment.

The inverted structure (p-i-n) has the opposite configuration, starting with the HTM material on the front electrode, perovskite, ETL, and the back metal electrode. Inorganic HTLs such as NiO_x_, CuI, or CuSCN claim higher chemical stability than the organic ones and are suitable choices for HTM material in this type of structure [18,19,20]. On top of perovskite, generally the ETL is made by combining C60 and BCP (bathocuproine) [18,19,20,21]. In the end, copper or silver electrodes are evaporated on top of ETL to finalize the device.

Perovskite properties and stability are still under debate [22,23,24]. It is well-known that perovskite grants low fabrication costs because it can be easily processed through precursor solutions with respect to Si or CIGS technologies [25,26]. Despite this, device stability is still an open issue because of the sensitivity of the photoactive material to humidity and micro-structure defects [6,23,24,27]. Moreover, the low stability of the large part of the organic molecule used as HTM and their dopants [28,29,30] and the diffusion of metal atoms from the electrode into perovskite are still open issues [31]. Different strategies are adopted to sort the problems related to perovskite [32,33,34,35,36,37] and HTM instability [38,39], but few works have focused on issues related to the metal counter electrode problem. As mentioned before, the most used counter electrode for perovskite devices is made of a high-cost metal like gold or silver. In this case, the deposition process requires an ultra-vacuum condition (10^−6^/10^−7^ bar), increasing the costs and making the process scalability arduous. Moreover, stability of the device will be compromised due to the ion migration of metal atoms from the back contact into the transport and active layers. These are the major reasons why metal electrodes have a huge CO_2_ footprint and cannot be considered the first choice for sustainable perovskite industrialization [40]. Despite this, metal electrodes grant better efficiencies compared to other types of counter electrodes. 

Carbon-based perovskite technology is a concept developed to improve the stability of PSCs and to lower the cost of the manufacturing process of PSCs. This material was used in DSSCs as an electrode for the first time and later applied also in solid-state photovoltaics [41,42,43,44,45,46]. Carbon-based electrodes are widely applied in PSCs because of their chemical inertness, hydrophobic nature, thermal stability, and compatibility with upscalable deposition techniques (e.g., screen-printing, blade-coating), signifying their solid potential for mass production [47,48,49,50,51,52,53,54]. Moreover, the approximate work function of 5.0 eV makes carbon a convenient counter electrode material for perovskite solar cells [55,56]. Here, we want to present a short overview of carbon-based devices and their manufacturing processes, looking at the different upscaling strategies that are the core of this emerging technology.

## 2. Materials and Methods

### 2.1. Carbon Electrodes

In PSCs, carbon-based electrodes demonstrated the ability to extract photogenerated holes from perovskite by themselves, opening the opportunity for researchers to explore HTL-free monolithic perovskite solar cells [57,58,59]. The control of graphite and carbon amounts inside precursor paste provides the optimal electrical properties according to the final device. Depending on which binder is used to realize the paste, a carbon electrode could be obtained by high-temperature (500 °C) or low-temperature curing (generally ≤ 120 °C) [60,61]. 

#### 2.1.1. High-Temperature Carbon Electrodes (HTCEs)

**Figure 2:**
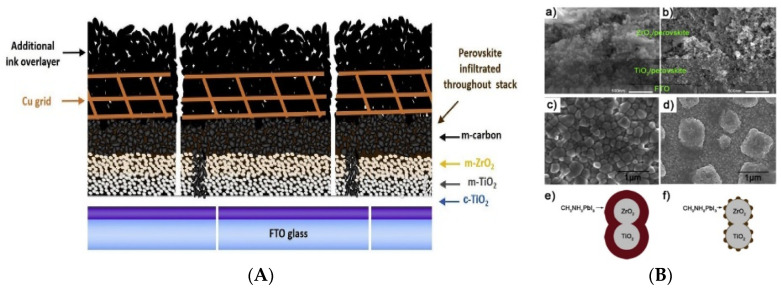
(**A**) Stack configuration with Cu metal grids; (**B**) SEM top view and cross section of FTO/TiO_2_/ZrO_2_/PVK/Carbon and illustration about perovskite poor filling issue. Figure 2A,B is reprinted with permission from references [70,72], respectively.

High-temperature carbon electrodes (HTCEs) are the first ones used in perovskite photovoltaics [62]. The core of the cell architecture is based on a fully mesoscopic structure made of three highly porous layers, called the triple-mesoscopic configuration. After the deposition of the anatase mp-TiO_2_ layer, a porous ZrO_2_ or Al_2_O_3_ spacer insulator layer avoids recombination and shunts with respect to the porous carbon counter electrode. All porous layers need high-temperature curing, about 400–500 °C. Perovskite is added by percolation through the carbon electrode by different coating techniques [62,63,64,65]. The easy fabrication of fully-printable triple-mesoscopic carbon-based (TiO_2_/ZrO_2_ or Al_2_O_3_/carbon) perovskite solar cells has gained widespread attention due to the exceptional stability and strong upscaling potential of such devices [62,63,66]. The first application of the triple-mesoscopic device was reported in 2013 with a power conversion efficiency (PCE) of 6.6% [56]. Since then, substantial improvements in performance have been made to generate a PCE above 17% [67,68]. Despite their good qualities, triple-mesoscopic carbon perovskite solar cells show different issues, such as the complete removal of solvent from perovskite percolated into the mesoscopic structure, the thickness of spacing layers, the poor filling of perovskite through a very thick carbon film (20–40 µm), and the photo-absorber morphology [69,70,71]. Moreover, the absence of any selective contact for photogenerated holes causes recombination pathways at the interface with the perovskite. In this context, Raptis et al. implemented metallic grids inside the carbon layer for better conductivity (Figure 2A) [72]. The stack utilized is TCO/TiO_2_/ZrO_2_/carbon where 25 µm of copper grids were applied between two layers of carbon. On the same stack configuration, Jiang et al. worked on a precursor solution concentration, demonstrating how a systematic study on perovskite precursor solutions led to an efficiency above 16% [67]. Liu et al. found critical parameters in the layer’s thickness that can improve the efficiencies and avoid poor filling issues of the full printable stack (Figure 2B) [70]. Liu et al. added a selective mesoporous layer of NiO_x_ as an HTL in a FTO/TiO_2_/Al_2_O_3_/NiO_x_/carbon device configuration. NiO_x_ accelerates the extraction of photogenerated holes and improves photovoltaic performance up to 17.02% [68]. In the literature, there are many approaches to solve the efficiency gap with respect to gold-based devices. The main strategies are focused on the charge extraction through hole Fermi level shift, the increase in carbon electrode conductivity, optimization of precursor solutions, and thickness of all the involved layers [67,68,70,72,73,74]. Despite these improvements, the high environmental and cost impact related to the high-temperature processes and non-radiative losses induced by the stack morphology reduce the feasibility on an industrial scale [40,75]. In addition, there are limited materials (e.g., inorganic HTM) compatible with high-temperature curing [39].

#### 2.1.2. Low-Temperature Carbon Electrodes (LTCEs)

**Figure 3:**
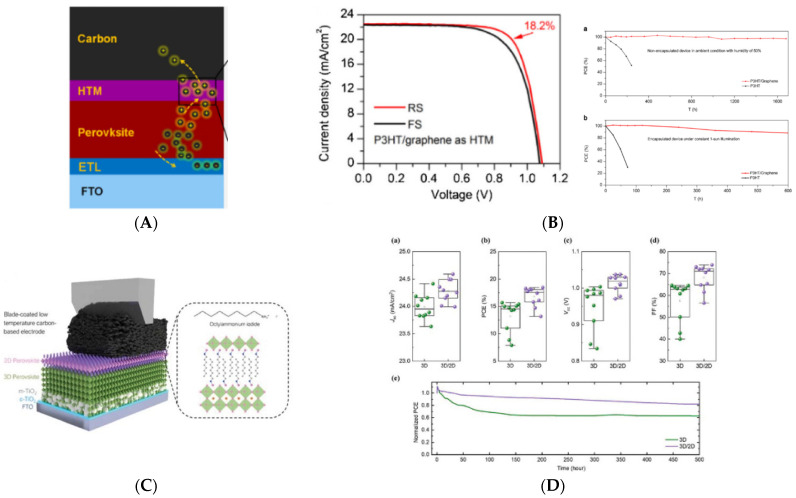
(**A**) Stack configuration; (**B**) Best JV curve (reverse and forward), shelf life and light-soaking stability for PSCs with P3HT/graphene as HTM using LTCE; (**C**) Stack; (**D**) Statistics on photovoltaic parameters and light-soaking test in nitrogen without encapsulation for 3D perovskite and 3D/2D perovskite carbon-based devices. All images are reprinted with permission from reference [76] (Figure 3A,B) and reference [92] (Figure 3C,D).

In the recent years, low-temperature carbon electrodes (LTCEs) in PSCs have earned a lot of attention for their low-cost manufacturing and long-term stability [76,77]. Moreover, improving the binder formulation to realize the carbon paste or ink makes it possible to obtain a good film with low-temperature annealing processes. LTCEs are used both in n-i-p (mesoscopic or planar) and p-i-n configuration [50,56,78,79,80,81]. LTCEs grant selective holes transport, a good conductivity for external contact, thermal and chemical stability, and high hydrophobicity [78]. This type of counter electrode and its relatively low curing temperature permit a deposition directly on top of the perovskite, without the poor filling and morphology control issues of HTCEs. Furthermore, organic/inorganic HTM can be used to achieve better charge extraction and efficiencies. The first attempt to make a device with LTCEs was made by Wei et al. by directly printing the carbon ink with perovskite, without any HTM as an interlayer, with an efficiency above 11% [82]. With the growth in interest in these materials, many techniques and optimizations were implemented in C-PSCs, including HTMs between PVSK and carbon electrodes and phase engineering of active materials. Recently, the incorporation of graphene-doped P3HT as an HTM in C-PSCs shows excellent PCEs above 18% (Figure 3A,B), with a shelf life and light-soaking stability of 1600 and 600 h, respectively [76]. Formamidinium-based perovskite coupled with Spiro-OMeTAD and pressed LTCE foils demonstrate the biggest efficiency for these devices, above 20% with 1000 h of shelf life stability [83]. Metal phthalocyanine [84,85], CuSCN [86], P3HT/NiO_x_-CNT [76,87], NiO nanoparticles [88], Cu_2_ZnSnS_4_ [89], and TPDI [90] are new relevant examples of HTMs employed with a low-temperature carbon electrode. Lately, low-dimension perovskite layers employing large cations like phenethyl ammonium iodide (PEAI) and octyl ammonium iodide (OAI) were used on top of 3D perovskite with LTCEs for interfacial engineering and 2D perovskite growth, with promising efficiencies of 15.6 and 18.5% (Figure 3C,D), respectively [91,92]. Calabrò et al. show the thermal (85 °C) stability improvement of a KI-doped perovskite cell by substituting the gold counter electrode with an LTCE [93]. He et al. doped the carbon paste with a small amount of CuPC as a p-dopant to modify the work function of the electrode and improve the band gap alignment. The PCE enhanced by 10% with respect to the bare carbon (from 12.8% to 14.8% for the recorded cell), and the stability of encapsulated devices reached 1000 h under continuous one-sun illumination at 45°C [94]. In recent years, LTCEs are also coupled with flexible substrates in perovskite solar technology, as shown by Babu et al. incorporating a thin layer of chromium between PCBM and carbon, achieving an efficiency above 15% and remarkable stability over 1000 h in shelf life at 85°C in a nitrogen environment [77].

### 2.2. Methodologies

**Figure 4:**
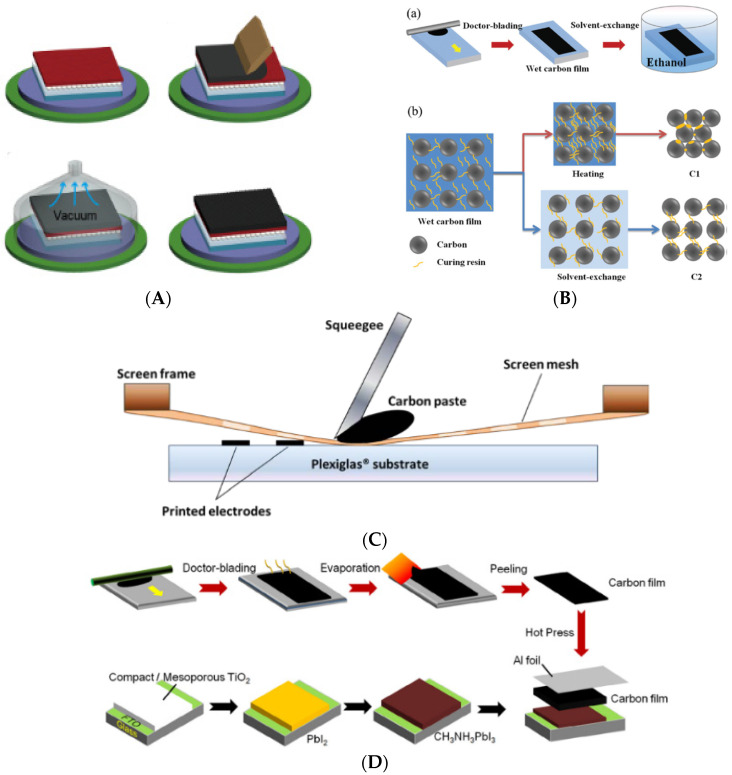
(**A**) Blade-coating deposition of carbon material and vacuum/heat treatment for fast drying the film; (**B**) Solvent exchange used to obtain carbon film; (**C**) Schematic structure of carbon paste printing with screen-printing technique; (**D**) Mechanical peeling-off of carbon film and incorporation into the solar device. Figure 4A,B is reproduced from references [98,100], respectively. Figure 4C,D is reproduced from references [102,103], respectively.

The upscaling of perovskite photovoltaic technology from small-area cells to modules and the related industrial and economical transition are achievable by scalable manufacturing processes, module design, and interconnection patterning [26,95,96]. The first point is related to the deposition of the full stack by scalable deposition techniques. In the literature, many efforts are related to the deposition of the perovskite layer to get homogeneous and highly efficient modules [97]. The carbon-based devices give the chance to deposit the full stack by printing techniques. Here, we just mention the most popular scalable deposition technique to obtain HT and LT carbon electrodes (Figure 4A–D). The blade-coating/doctor-blading technique deposits material using a blade on a rigid or flexible substrate. Highly homogeneous films are obtained by modifying the material amount, the meniscus gap, and the concentration and composition of precursor solutions. After deposition, an annealing process is required, sometimes in a vacuum chamber to make more efficient the solvents’/binders’ evaporation [98]. The screen-printing technique adopts a patterned screen to deposit the material with a controlled thickness. These scalable deposition methods could be implemented in manufacturing processes such as roll-to-roll [70,84,90,99]. Press transfer and hot press are alternative methods to obtain single-carbon film on the substrate [100,101,102]. These types of techniques avoid the annealing process and preserve organic HTM or the passivating agent on top of the perovskite. In this case, the carbon film is obtained by printing techniques; then, with solvent exchange or mechanical peeling-off, the film is picked up from the substrate and mechanically implemented through a press onto the solar device.

Though spin-coating, ink-jet printing, and spray-coating techniques are less used to obtain carbon counter electrodes because of the adopted viscous binders (e.g., ethylene glycol), in the literature, different examples are presented exhibiting competitive efficiencies [104,105,106]. 

## 3. Upscaling Carbon-Based Perovskite Technology

One of the main goals achievable with carbon-based perovskite technology is the possibility to have an upscaled process for the full stack. Carbon material combines good electrical properties with easy manufacturing processes thanks to the optimization of carbon-paste formulations and related deposition methods. These features together with the high stability make carbon a real candidate to supplant costly metal electrodes in perovskite technology. Here, we report the main results obtained on module devices working with HTCE and LTCE (Table 1).

### 3.1. Upscaling of High-Temperature Carbon Electrodes and Perovskite Devices

**Figure 5:**
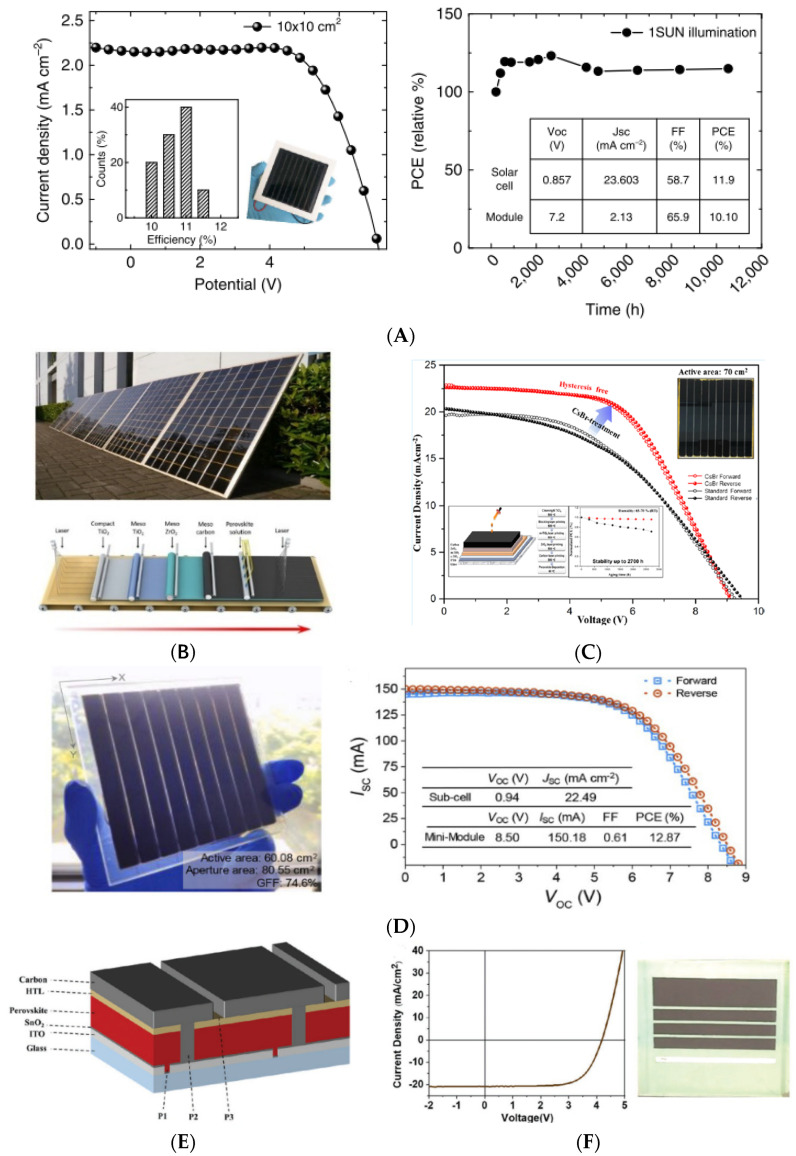
(**A**) JV curve of 2D/3D perovskite with 3% AVAI in 10 × 10 cm^2^ module and module stability test under 1 sun; (**B**) 7 m^2^ printable perovskite solar panels with HTCE and illustration of ideal product line; (**C**) JV curves with and without CsBr-modified TiO_2_ solar HTCE module and shelf life stability (inset); (**D**) Picture and JV curve of HTCE solar module with slot-die coating perovskite infiltration; (**E**) Module stack; (**F**) JV curve and picture of low-temperature carbon module on 25 cm^2^ substrate using P3HT as HTM. Figure 5A,B is reproduced with the permission from references [63,108], respectively; Figure 5C,D is reproduced from references [110,111], respectively. Figure 5E,F is reproduced with permission from reference [83].

In 2013, Ku et al. showed the first HTCE heterojunction perovskite solar cell by a printable deposition method with an efficiency of 6.64% [62]. Over the last 9 years, many large-area perovskite modules working with carbon counter electrodes were reported in the literature. In 2016, Priyadarshi et al. reported a monolithic perovskite module with an active area of 70 cm^2^, PCE of 10.74%, and ambient stability of more than 2000 h. The meso-TiO_2_/ZrO_2_/carbon stack was deposited by the screen-printing technique, and the perovskite absorber was obtained by the drop-casting method [107]. Grancini et al. demonstrated the stability of a 5-AVAI/MAPI perovskite solar module with 2D/3D perovskite interface engineering [108]. The fabrication of 10 × 10 cm^2^ solar modules by a fully printable industrial-scale process reached 11.2% efficiency with zero loss at 1 sun AM 1.5 G, 55 °C for 10,000 h (Figure 5A) [108]. 5-AVAI is also used by Hu et al. combined with γ-butirrolactone (GBL) to control perovskite solvent evaporation after the infiltration step. The authors presented a 7 m^2^ solar panel fully fabricated with printable techniques (Figure 5B) [63]. De Rossi et al. use 5-AVAI/MAPI perovskite in a triple-mesoscopic module with a 198 cm^2^ active area and an efficiency of 6.6% after storing the module in the dark at 50–70% RH [109]. Thus, they show how the patterning optimization of the blocking layer could improve device performances. More recently, a modified mesoporous scaffold with CsX (X = halide, Figure 5C) salts showed a boosted open circuit voltage of 960 mV with respect to the reference 920 mV on cells (0.7 cm^2^ active area) and modules (70 cm^2^) with a PCE of 12.59% and 11.55%, respectively, and stability over 2000 h in ambient conditions [110]. Xu et al. present 60.08 cm^2^ active area module with a controlled infiltration method by slot-die coating. The control of the precursor solution and deposition of perovskite above the triple-mescocopic scaffold grant a final PCE of 12.87%, which is the highest value reported for such large devices (60 cm^2^) [111]. Recently, Kobayashi et al. reported an evaluation about the stability of HTC-based devices. They show 4.32 cm^2^ active area modules with a triple-mesoscopic high-temperature stack (meso-TiO_2_/ZrO_2_/carbon) [112]. The module had a PCE equal to 8.7% and was stable in damp-heat aging conditions (85 °C/85% RH); it showed stability for more than 3000 h. This stability is attributed to the light-induced performance-increasing phenomenon. The mechanism is associated to the organic molecules 5-ammoniumvaleric acid and methylammonium forming a quasi-2-dimensional perovskite/metal oxide interface with a positive effect on charge transport and suppression of ion migration [112]. 

### 3.2. Upscaling of Low-Temperature Carbon Electrodes and Perovskite Devices

In the literature, few works are present investigating LTCE-based module fabrication. In 2017, Cai et al. coupled gas-pumping perovskite with a slot-die-coating method and a low-temperature carbon electrode [113]. This approach led to reproducible solar modules (FTO/ZnO/PVK/carbon) with 17.6 cm^2^ active area, a PCE of 10.6%, and no significant degradation after 140 days of outdoor testing [114]. In 2019, He et al. reported a full low-temperature-processed n-i-p mesoscopic module by doping the carbon counter electrode with copper (II) phtalocyanine. The aperture area was 22.4 cm^2^, the geometrical fill factor was 89.6%, and the efficiency was 7.2% [94]. In 2021, Yang et al. added 10% guanidinium chloride (CH_6_N_3_^+^Cl^−^) to the perovskite precursor solution and used P3HT as an HTM (Figure 5E,F) [83]. The morphology and crystallinity of perovskite film were greatly enhanced, resulting in enlarged grain sizes and lowered defect densities. Moreover, they studied the passivation of the active layer with PDCBT and the interface engineering between HTM and carbon with Ta-WO_x_. All these optimizations led to amazing efficiency of 15.3% on a 4 cm^2^ module with a fully printable fabrication process.

## 4. Conclusions and Perspectives

In just a few years, state-of-the-art perovskite solar cells and modules have reached impressive efficiency. Metal electrodes are intensively used despite the high costs of the materials and processes. The future of photovoltaic energy cannot base its growth on high-cost materials with a high CO_2_ footprint and energy consumption. Carbon-based materials are processable with scalable techniques at high and low temperature as PSC counter electrodes. In the literature, we found different approaches for improving and scaling up HTCE on PSM technology both on modules and panels. Fully printable triple-mesoscopic devices are a suitable choice for their easy adaptability in a possible pilot line production, but despite the excellent stability, huge differences in efficiency are still present for such devices with respect to the metal-based perovskite cells. LTCE grants low-temperature processing with relatively less energy costs. Moreover, it is possible to use various HTMs as a selective contact between perovskite and carbon material, retaining perovskite morphology as well. All these features will drive the fabrication of low-temperature carbon-based solar modules with high efficiency and stability in the coming years. Nowadays, there is a lack of literature about LTCE-based modules. Many efforts should be oriented towards scalable processes for such devices starting from the laser interconnection patterning, which has not been deeply detailed or explored. Low-temperature carbon-based PSCs have the chance to be the perovskite solar technology ready and suitable for the market. The efficiency is above 20%, and the research community is shifting its attention in this field away from the expensive and pollutant metal-electrode-based PSCs. Here, we have reported the state of the art on HT and LT carbon-based perovskite modules to help researchers understand the most feasible way towards an efficient, stable, and sustainable solar device.

## Figures and Tables

**Table 1:** Reported deposition technique of carbon electrode, photovoltaic parameters, stability tests, and device stack configuration on perovskite solar module with high- and low-temperature carbon electrode.

	Deposition Method of Carbon Layer	Module Active Area	Voc [V]	Isc [mA]	FF [%]	PCE [%] (fwd)	Stability Tests	Stack Configuration	Ref.
**High-Temperature carbon modules**	Screen-printing	49 cm^2^ (10 cells)	9.3	98	56	10.4 (10.4)	(1) 1000 h light soaking at 1 sun (2) 30 days outdoor test (3) 1 year shelf life in dark	FTO/c-TiO_2_/mTiO_2_/mZrO_2_/mCarbon/ (5-AVAI)_x_MA_1-x_PbI_3_	[63]
	Screen-printing	70 cm^2^ (10 cells)	9.63	124	63	10.74 (10.10)	2000 h shelf life	FTO/c-TiO_2_/mTiO_2_/mZrO_2_/mCarbon/ (5-AVAI)_x_MA_1-x_PbI_3_	[107]
	Screen-printing	47.6 cm^2^ (7 cells)	7.05	104.7	70	11.16 (-)	12.000 h at 1 sun AM 1.5 G conditions at 55 °C	FTO/c-TiO_2_/mTiO_2_/mZrO_2_/mCarbon/(AVA)_x_(MA)_1-x_PbI_3_	[108]
	Screen-printing	198 cm^2^ (22 cells)	19.7	192	34	6.6 (5.7)	-	FTO/c-TiO_2_/mTiO_2_/mZrO_2_/mCarbon/(5-AVAI)xMA1-xPbI_3_	[109]
	Screen-printing	70 cm^2^ (10 cells)	9.12	158.3	56	11.55 (11.24)	2700 h shelf life stability on small area cells at 60% RH	FTO/cTiO_2_/mTiO_2_/mZrO_2_/mCarbon/(5-AVAI)_x_MA_1-x_PbI_3_	[110]
	Screen-printing	60.08 cm^2^ (9 cells)	8.50	150.1	61	12.87 (-)	-	FTO/cTiO_2_/mTiO_2_/mZrO_2_/mCarbon/(5-AVAI)_x_MA_1-x_PbI_3_	[111]
	Screen-printing	4.32 cm^2^ (3 cells)	2.4	10.8	34	8.7 (8.7)	3000 damp-heat 85 °C/85 RH	FTO/cTiO_2_/mTiO_2_/mZrO_2_/mCarbon/(5-AVAI)_x_MA_1-x_PbI_3_	[112]
**Low-Temperature carbon modules**	Doctor-blade	22.4 cm^2^	6.4	53	47.5	7.2 (-)	-	FTO/c-TiO_2_/MAPI/Carbon doped with CuPC	[94]
	Screen printing	17.6 cm^2^ (8 cells)	6.14	57.2	53	10.6 (-)	-	FTO/ZnO/MAPI/Carbon	[114]
	Blade-coating	4 cm^2^ (Electrical values referred to sub-cell)	1.05	21.2	69	15.3 (-)	800 h at 85 °C in nitrogen (on small area cells) 180 s MPP tracking on module	ITO/SnO_2_/GA_x_MA_1-x_PbI_3_/PDCBT/P3HT/Ta-WOx/Carbon	[83]

## Data Availability

Not applicable.
